# Real-World Observational Analysis of Clinical Characteristics and Treatment Patterns of Patients with Chronic Sialorrhea

**DOI:** 10.3390/toxins16080366

**Published:** 2024-08-17

**Authors:** Michael A. Hast, Amanda M. Kong, Jenna Abdelhadi, Rohan Shah, Andrew Szendrey, Jordan Holmes

**Affiliations:** 1 Merz Therapeutics, Raleigh, NC 27615, USA; jordan.holmes@merz.com; 2 Aetion, New York, NY 10001, USA; amanda.kong@aetion.com (A.M.K.); jennahadi2@gmail.com (J.A.); rohan.shah@aetion.com (R.S.); ayeszendrey@gmail.com (A.S.)

**Keywords:** anticholinergic, botulinum toxin, sialorrhea, treatment utilization, neurologic disorders, Parkinson’s, cerebral palsy

## Abstract

Chronic sialorrhea is a condition characterized by excessive drooling, often associated with neurological and neuromuscular disorders such as Parkinson’s disease, cerebral palsy, and stroke. Despite its prevalence, it remains underdiagnosed and poorly understood, leading to a lack of comprehensive data on patient demographics, clinical characteristics, and treatment patterns. This study aimed to help fill these existing gaps by analyzing real-world data using Optum’s de-identified Clinformatics^®^ Data Mart Database. Patients were required to have a diagnosis indicative of sialorrhea plus evidence of sialorrhea treatment between 1/1/2007 and 5/31/2022. Two cohorts were analyzed: patients with evidence of newly diagnosed sialorrhea and associated treatment, and sialorrhea patients initiating incobotulinumtoxinA. Clinical characteristics, comorbidities, symptoms, and treatment utilization were described before and after diagnosis and incobotulinumtoxinA initiation. No formal statistical comparisons were performed. Patients were predominantly aged 65 or older, male, and non-Hispanic white. Parkinson’s disease and cerebral palsy were the most common comorbidities among adults and children, respectively. Treatment patterns suggest that anticholinergics are more commonly used than botulinum toxin therapy. The findings offer valuable information for improving diagnosis and treatment approaches and suggest a need for further research into treatment effectiveness, safety, and disease burden.

## 1. Introduction

Chronic sialorrhea, characterized by excessive drooling beyond the lip, poses a challenge for both patients and clinicians alike. It is a prevalent issue among neurologically impaired individuals across various age groups, although it can also occur independently [1,2]. This condition often manifests as a symptom of neurological or neuromuscular disorders such as Parkinson’s disease, cerebral palsy, traumatic brain injury, amyotrophic lateral sclerosis, and stroke [3]. Chronic sialorrhea can profoundly impact a patient’s quality of life due to associated complications such as perioral skin damage, speech impairments, and social stigmatization [3]. Additionally, it poses a risk of dysphagia and aspiration pneumonia in certain neurologically impaired populations, which creates a clinical danger for these patients [3].

Despite its prevalence and clinical significance, chronic sialorrhea remains a poorly understood and often underdiagnosed condition. Current treatment options for chronic sialorrhea range from more conservative measures such as lifestyle modifications or pharmacologic interventions to more aggressive approaches, such as surgical excision of the salivary gland [4]. Historically, oral anticholinergics have been the first-line therapy of choice for providers, as medications such as glycopyrrolate and scopolamine inhibit the muscarinic receptors on the salivary glands, thereby inhibiting salivary production. In particular, glycopyrrolate (Cuvposa) is FDA-approved for pediatric patients with chronic severe drooling. However, due to their systemic effects, anticholinergics often result in unwanted side effects such as an excessively dry mouth, constipation, and cognitive impairment, which may limit patient adherence and long-term use [4,5,6]. 

More recently, botulinum toxin (BoNT) therapy has shown considerable promise in the management of chronic sialorrhea. IncobotulinumtoxinA (Xeomin) has been approved by the FDA for both adults and children for this indication, whereas rimabotulinumtoxinB (MYOBLOC) has received approval only for adults. However, onabotulinumtoxinA (Botox) is frequently employed off-label for this purpose [5]. Overall, BoNT injections have demonstrated effectiveness and good tolerability in clinical studies while also foregoing many side effects often seen with anticholinergic therapy [7,8,9].

To date, few studies characterizing the chronic sialorrhea patient journey exist; further, no real world data (RWD) studies have been conducted in this population. In order to fill this gap, this study’s primary objective was to provide a comprehensive description of the demographics, clinical characteristics, and treatment patterns among patients exhibiting evidence of chronic sialorrhea. The secondary objective was to describe the utilization of incobotulinumtoxinA and treatment patterns prior to its initiation as the only approved therapy for both children and adults in the United States. A large, geographically diverse health insurance claims database, Optum’s de-identified Clinformatics^®^ Data Mart Database (CDM), was chosen for this analysis to describe a large number of patients and to account for possible regional variations in treatment.

## 2. Results

### 2.1. Primary Objective

#### 2.1.1. Results Summary

Sialorrhea patients were predominantly aged 65 or older, male, and non-Hispanic white. Among adults, Parkinson’s disease was the most common comorbidity while cerebral palsy was the most common comorbidity among the pediatric population. Anticholinergic medications were more commonly used than BoNT, with over two-thirds of patients having a claim for an anticholinergic medication during follow-up.

#### 2.1.2. Demographic Characteristics of Sialorrhea Patients

**Figure 1:**
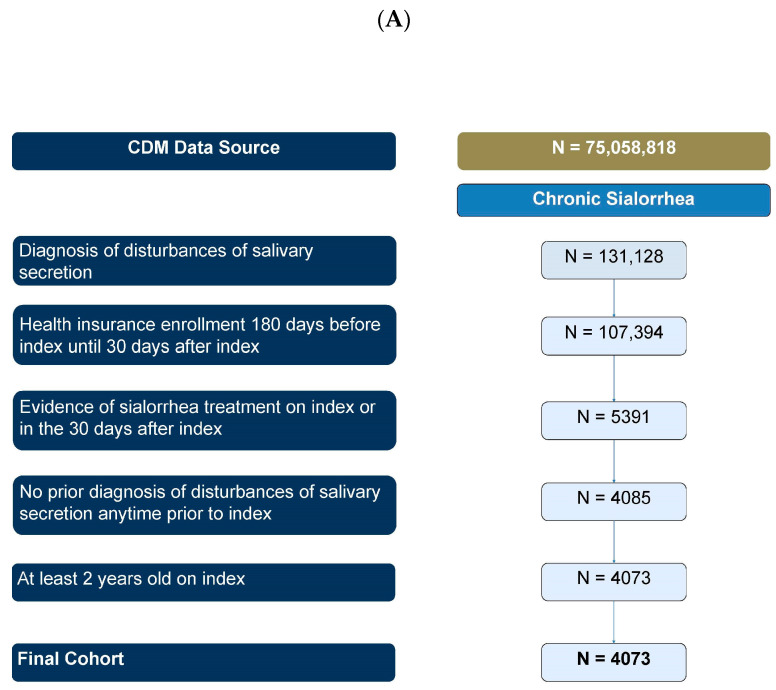

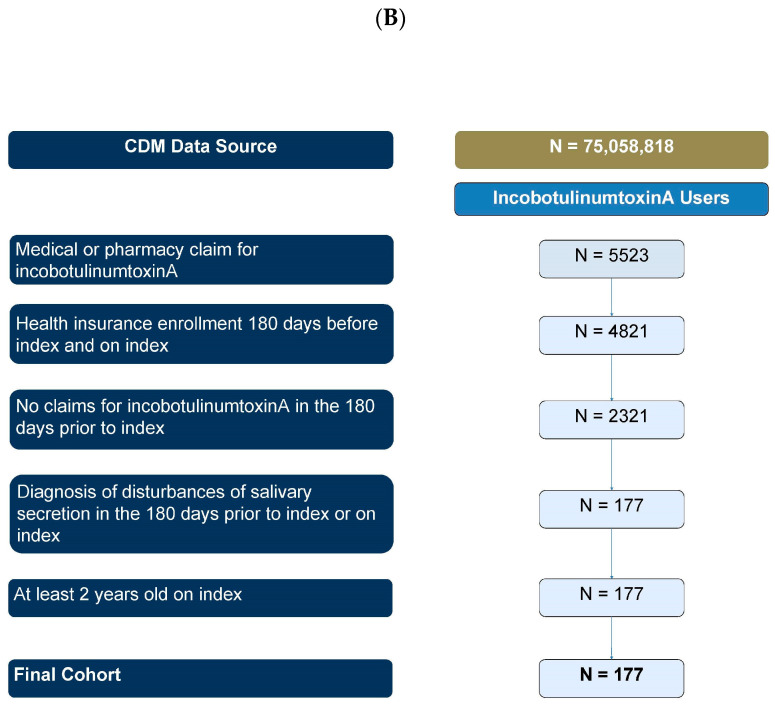
(**A**,**B**) Patient attrition of overall chronic sialorrhea cohort and incobotulinumtoxinA cohort.

For the primary objective, a total of 4073 sialorrhea patients were identified (Figure 1A,B), with a median age of 10 years (IQR: 5, 13) among pediatric patients and 68 years (IQR: 56, 78) among adult patients. The population was predominantly male (54.6%) and non-Hispanic white (67.2%), with consistent sex and geographic distributions between pediatric and adult strata. A majority of patients resided in the South (42.3%), while the fewest were in the Northeast (12.6%), which is reflective of the data source. All pediatric patients were commercially insured, while the majority of adult patients were insured through Medicare (70.7%) (Table 1).

#### 2.1.3. Clinical Characteristics and Treatments before and after Sialorrhea Diagnosis

The study revealed an increase in the frequency of all comorbidities and clinical symptoms between baseline and follow-up periods for both pediatric and adult patients (Table 2). Among pediatric patients, cerebral palsy was the most common comorbidity, affecting 56.0% to 67.6% of patients between baseline and follow-up. In adults, Parkinson’s disease was the most frequently reported comorbidity, with an increase from 19.3% in baseline to 26.0% in follow-up, followed by stroke, which increased from 11.4% at baseline to 17.3% in follow-up. The most prevalent clinical symptom observed was dysphagia, which increased from 36.4% at baseline to 54.0% in follow-up, consistently affecting both pediatric and adult patients.

A total of 3874 patients had a new claim for any treatment at any time during follow-up, with a median time to treatment of 3 days (IQR: 0.5, 16). Of note, many of the therapies described here may be used for conditions other than chronic sialorrhea; therefore, the results should be interpreted with that context in mind. Among these patients, 2827 had a claim for anticholinergics, 1389 for a BoNT, and 13 for surgical excision of salivary glands. Additionally, 199 patients had a treatment claim prior to their sialorrhea diagnosis that overlapped with their follow-up period, rather than a new claim during follow-up.

Prior to their sialorrhea diagnosis, 37.4% of patients had a claim for anticholinergics at baseline, which increased to 69.4% in follow-up. For patients with a claim for anticholinergics in follow-up, the median time to treatment was 6 days (IQR: 0.5, 20). Glycopyrrolate and scopolamine were the most commonly used anticholinergics (32.7% and 32.1% in follow-up, respectively). Glycopyrrolate was more commonly used in pediatric patients (73.4% in follow-up), while scopolamine was more commonly used in adults (33.2% in follow-up).

BoNT therapies were less frequently used than anticholinergics, with any BoNT use increasing from 7.5% at baseline to 34.1% in follow-up. The median time to BoNT treatment was 0.5 days (IQR: 0.5, 20), shorter than the time to anticholinergic treatment. OnabotulinumtoxinA was the most frequently used BoNT (6.6% at baseline, 21.9% in follow-up), followed by rimabotulinumtoxinB (0.4% at baseline, 13.0% in follow-up). IncobotulinumtoxinA use also increased from 0.2% at baseline to 2.3% in follow-up. Among the 1389 patients with BoNT therapy, the majority (88.8%) had a claim for only one BoNT formulation.

Of the 2827 patients with an anticholinergic claim, 10.9% switched to BoNT therapy after their anticholinergic therapy, which was more common in pediatric patients (25.9%) than adults (8.9%). Among patients with a BoNT claim, 20.3% switched to anticholinergic therapy after their BoNT claim, with pediatric patients more likely to switch (60.5%) compared to adults (15.4%).

#### 2.1.4. Treatment Patterns of Sialorrhea Patients in Follow-Up

For the purpose of assessing treatment patterns in follow-up, treatments were considered only if they had a minimum duration of 30 days (Table 3). A total of 3241 patients had any treatment claim during the follow-up period. The predominant first-line treatment was anticholinergic therapy, accounting for 62.1% of first-line treatments. BoNT therapies were also used as first-line treatments, with onabotulinumtoxinA representing 20.1%, rimabotulinumtoxinB 13.2%, and incobotulinumtoxinA 1.6%. Additionally, 1270 patients received a second-line treatment, of which the majority were treated with anticholinergics (54.3%), followed by onabotulinumtoxinA (20.1%). Among the 661 patients who advanced to a third-line treatment, 54.8% received anticholinergics and 19.5% received onabotulinumtoxinA. These treatment patterns were consistent across both pediatric and adult patient populations. The proportions of patients with specific BoNT formulations are suppressed due to small counts in some cases.

### 2.2. Secondary Objective

#### 2.2.1. Results Summary

Users of incobotulinumtoxinA with sialorrhea were predominately over 75 years of age, male, and non-Hispanic white. Nearly two-thirds of patients had a diagnosis code for Parkinson’s disease. A small proportion of patients had evidence of anticholinergic use after initiating incobotulinumtoxinA or evidence of another formulation of BoNT after incobotulinumtoxinA treatment.

#### 2.2.2. Demographic Characteristics of IncobotulinumtoxinA Users

Due to the small number of pediatric patients (<10), results were not stratified by age group in this analysis. A total of 177 incobotulinumtoxinA users were identified (Figure 1B). These patients were predominantly aged 65 years or older (75.7%), male (62.1%), and non-Hispanic white (73.4%), making them slightly older than the overall sialorrhea population. All patients had commercial (22%) or Medicare (78%) insurance, with a slight skew toward the South (36.2%) and West (31.6%) regions, reflective of the data source (Table 1).

#### 2.2.3. Clinical Characteristics and Treatments before and after IncobotulinumtoxinA Injection

Parkinson’s disease was the most common comorbidity observed, occurring in 62.1% to 65.0% of patients, a much higher frequency compared to the overall sialorrhea population (17.4% to 23.5%) (Table 2). The second and third most common diagnoses during these periods were stroke (8.5% to 16.4%) and Alzheimer’s disease/dementia (6.2% to 14.1%). Common clinical symptoms included dysphagia (37.3% to 53.1%), depression (22.6% to 30.5% in follow-up), anxiety (15.3% to 22.0%), and dehydration (20.9% to 2.3%).

Prior to the initiation of incobotulinumtoxinA treatment, 26% of patients had a claim for a non-incobotulinumtoxinA toxin. The use of rimabotulinumtoxinB increased slightly from baseline to follow-up (from 14.7% to 15.3%), while the use of onabotulinumtoxinA decreased (from 11.3% to 8.5%). In follow-up, 19 patients (10.7%) had evidence of concurrent use of a BoNT and anticholinergic therapy, with a median overlap of 75 days (IQR: 47, 253).

#### 2.2.4. IncobotulinumtoxinA Utilization 

The median follow-up time for incobotulinumtoxinA users was 1.01 years (IQR: 0.48, 2.37), with a median number of injections per person of 1 (IQR: 1, 3) (Table 4). Approximately 49.2% of patients had more than one injection, with a median duration of treatment until discontinuation or switch of 113 days (IQR: 99, 226). Switching to a non-incobotulinumtoxinA BoNT occurred in 22.6% of patients, with a switch to rimabotulinumtoxinB being more common than onabotulinumtoxinA (14.3% vs. 8.5%). Neurology providers administered the majority (57.2%) of incobotulinumtoxinA injections.

## 3. Discussion

This descriptive study sheds light on the burden of chronic sialorrhea among patients in the United States, revealing high rates of comorbidities, clinical symptoms, and treatment use. Despite not all patients having a new treatment claim during follow-up, a significant proportion utilized long-term therapies such as anticholinergics. To our knowledge, this is one of the first RWD analyses focusing on this frequently underdiagnosed and undertreated condition. However, the findings are consistent with the available information on the most common etiologies and treatment modalities [5]. It has been reported that over 80% of patients with Parkinson’s disease experience sialorrhea [10]. We found Parkinson’s disease to be the most common comorbidity among adults with sialorrhea and incobotulinumtoxinA users. Similarly, over half of the pediatric patients analyzed here had a diagnosis of cerebral palsy. The prevalence of sialorrhea among children with cerebral palsy may be up to 58% [11]. 

Demographics and patient characteristics did not markedly differ between the overall sialorrhea cohort and incobotulinumtoxinA users. However, incobotulinumtoxinA users tended to be older and had a higher prevalence of Parkinson’s disease, suggesting that its utilization may be more common in patients with more advanced disease stages.

One notable finding is the widespread off-label use of anticholinergics among patients with chronic sialorrhea, since BoNT is the only approved treatment in adults. Despite this, anticholinergic therapy remains the predominant treatment modality in these patients, suggesting an awareness gap for the use of BoNT to treat sialorrhea, in addition to what is believed to be an underdiagnosis problem for the condition itself. Additionally, clinical trials for incobotulinumtoxinA and rimabotulinumtoxinB have reported some localized side effects such as injection site pain, bruising, and muscle weakness [7,8,9]. In contrast, anticholinergic medications can have systemic effects including constipation, blurred vision, urinary retention, and cognitive impairment [2,6,12]. It is noteworthy that unlike anticholinergics, which act systemically and may entail a range of side effects, BoNT therapy is localized, potentially yielding a more favorable side effect profile. Other considerations impacting treatment choice include provider preference and insurance coverage. 

This study has several strengths. There are few publications in the literature that outline the pathways of treatments for sialorrhea patients, and existing references primarily identify sialorrhea as a subset of patients diagnosed with various neuromuscular disorders [1,2,3,6]. This descriptive study provided further insight into the demographics of patients diagnosed with sialorrhea and the management of sialorrhea, particularly BoNT and anticholinergic usage. This study also provided clinical profiles that may have occurred in real-world practice that may not have been observed in controlled clinical trial settings. 

Moreover, there are several benefits afforded by conducting this study using the CDM. To our knowledge, this is the first descriptive study of sialorrhea patients utilizing an administrative claims data source. The data are collected during routine clinical care and provides a representative view of the underlying patient population’s treatment management. The inclusion of important patient demographics, including race, enabled this study to better characterize patients diagnosed with sialorrhea and the subset of patients being treated with incobotulinumtoxinA.

Nevertheless, this study has its limitations. Real-world data analyses are susceptible to biases and limitations inherent to claims data, such as potential misclassification of comorbidities and outcomes. Claims data are primarily collected for reimbursement purposes rather than clinical research, and therefore, reliance on diagnosis codes to identify comorbidities and outcomes for this study may lead to potential misclassification. The results described in this analysis may not be generalizable to other populations with different insurance coverage or uninsured patients. Regional variations in the use of BoNT may exist and sampling in CDM varies by state [13]. The identification of sialorrhea patients may be overestimated due to the use of the broad diagnosis codes for disturbances of salivary secretion, including both sialorrhea and dry mouth. Requiring the use of a sialorrhea treatment may improve the identification of patients. Similarly, because a diagnosis code specific to sialorrhea does not exist, patients who do not seek care or treatment were included in the analyses. It should also be acknowledged that sialorrhea may be underdiagnosed or undertreated as a result of physicians often considering sialorrhea a secondary condition to the patient. Exposure misclassification of incobotulinumtoxinA and non-incobotulinumtoxinA therapies should be minimal because botulinum toxins are administered by a healthcare provider, making them less susceptible to assumptions of medications that are self-administered, such as oral medications. However, patients may have been using incobotulinumtoxinA or other treatments for conditions other than chronic sialorrhea. This is also true of patients using non-incobotulinumtoxinA botulinum toxins as a diagnosis code, as disturbance of salivary secretion was not required to occur on the same day. The proportion of patients who switched also does not account for variable follow-up time. Patient-reported outcomes or other measures of effectiveness are not available in this data source. Explorations have indicated that units administered are also not reliably captured in this data source. 

Likewise, data on patient sociodemographic factors (e.g., socioeconomic status), patient-reported outcomes (e.g., quality of life indicators), or clinical details regarding disease status (e.g., severity, progression) and sequelae of disease (e.g., disability) are generally not included in claims databases. The data source includes race/ethnicity data collected from public records and uses an imputation method that employs validated algorithms incorporating racial and ethnic neighborhood composition as ascertained by the United States (US) Census, residential zip code, and first and last name [14]. Despite the potential for misclassification, research evaluating the validity of imputed race with similar methods has previously demonstrated moderate sensitivity (48%) in estimating the race of black participants, but higher specificity (97%). A comparison of race imputed by these algorithms with self-reported race also showed a positive predictive value of 71% in estimating the race of black participants [14]. It is important to note that patients in this study were followed for an average of one year. However, given that a large number of these patients are likely to have sialorrhea secondary to an underlying systemic disease, it is possible that their clinical characteristics and treatment patterns change over time as their disease progresses.

## 4. Conclusions

The results of this analysis can be used to better understand the patient journey of chronic sialorrhea patients, which may help improve diagnosis and treatment efforts in the future. Given the observed treatment patterns, there is a clear opportunity to raise awareness among healthcare providers regarding the benefits of initiating BoNT therapy in this patient population. Further research should include direct comparisons between anticholinergics and BoNT therapies in a group of newly treated patients to directly assess cost, treatment effectiveness, side effect profiles, and clinical endpoints such as the need for surgical excision. More studies are needed with larger samples and with longer follow-up periods, potentially conducted on other data sources such as electronic medical records with provider notes to refine the patient population and confirm which treatments were used for chronic sialorrhea versus other conditions. 

## 5. Materials and Methods

### 5.1. Data Source

This study utilized CDM. CDM is derived from a database of administrative health claims for members of large commercial and Medicare Advantage health plans. It utilizes medical and pharmacy claims to derive patient-level enrollment information, healthcare costs, and resource utilization information. The population is geographically diverse, spanning all 50 states, and is statistically de-identified under the Expert Determination method, consistent with HIPAA, and managed according to Optum^®^ customer data use agreements. CDM administrative claims submitted for payment by providers and pharmacies are verified, adjudicated, and de-identified prior to inclusion. Race/ethnicity data were obtained from public records and imputed using validated algorithms incorporating racial and ethnic neighborhood composition as ascertained by the US Census, residential zip code, and first and last name [14].

### 5.2. Study Design

An observational, retrospective cohort study was conducted using two non-mutually exclusive cohorts to address each study objective: (1) patients with incident sialorrhea and (2) patients with sialorrhea treated with incobotulinumtoxinA. Potential index dates ranged from 1 July 2007 to 31 May 2022, with the full study period extending from 1 January 2007 to 30 June 2022. Patients were followed from the index date until the earliest date of disenrollment in the health plan (allowing up to 45-day gaps), end of data, or death.

### 5.3. Primary Objective Patient Selection

For the primary objective, a primary cohort of newly diagnosed sialorrhea patients with any treatment was created. To be included in the primary cohort, patients were required to have a medical claim with an International Classification of Disease, Clinical Modification (ICD-9-CM or ICD-10-CM) diagnosis code in any position for disturbances of salivary secretion (ICD-9: 527.7 or ICD-10: K11.7) between 1 July 2007 and 31 May 2022, which was referred to as the index date. In addition, patients were required to have a medical or pharmacy claim with a Healthcare Common Procedure Coding System (HCPCS), National Drug Code (NDC), or generic name for a sialorrhea treatment indicating evidence of sialorrhea treatment coverage within the 30 days after the index date (inclusive of the index date). Patients could also satisfy the treatment requirement if they had evidence of a treatment that started prior to their sialorrhea diagnosis with drug coverage overlapping with index date. In addition, patients had to be at least 2 years old on the index date and have continuous health plan enrollment spanning from at least 180 days prior to the index date to 30 days after the index date, with an allowable gap of 45 days. Patients were excluded from this cohort if they had missing age or sex information on the index date or a medical claim with an ICD-9-CM or ICD-10-CM diagnosis code indicating disturbances of salivary secretion at any time prior to the index date.

### 5.4. Secondary Objective Patient Selection

For the secondary objective, a secondary cohort of sialorrhea patients with incobotulinumtoxinA use, indexed on the first use of incobotulinumtoxinA following a 180-day washout, was created. Patients were required to have a medical claim or pharmacy claim for incobotulinumtoxinA injection, which was referred to as the index date. Additionally, patients were required to have a medical claim with a diagnosis code in any position for disturbances of salivary secretion (ICD-9: 527.7 or ICD-10: K11.7) in the 180 days prior to or on the day of the qualifying incobotulinumtoxinA injection, be at least 2 years old on the index date, and have continuous health plan enrollment spanning from at least 180 days prior to the index date to 30 days after the index date, with an allowable gap of 45 days. Patients were excluded from the secondary cohort if they had missing age or sex information on the index date or a medical or pharmacy claim for incobotulinumtoxinA injection in the 180 days prior to the index date.

### 5.5. Patient Demographic and Clinical Characteristics

Patient demographics, including age, sex, race/ethnicity, region, and insurance type, were measured on the index date. The following comorbidities and symptoms were assessed during the 180-day baseline and follow-up periods via the presence of diagnosis codes on medical claims: Alzheimer’s disease/dementia, amyotrophic lateral sclerosis, anxiety, blepharospasm, cerebral palsy, cervical dystonia, dehydration, depression, halitosis, dysphagia, multiple sclerosis, Parkinson’s disease, speech impairment, stroke, traumatic brain injury, and upper limb spasticity.

### 5.6. Treatments

To determine treatment use, medical and pharmacy data were evaluated for the presence of procedure codes, NDCs, and generic names for the following treatments: botulinum toxins (incobotulinumtoxinA, rimabotulinumtoxinB, onabotulinumtoxinA), anticholinergic drugs (glycopyrrolate, benztropine, scopolamine, tropicamide, biperiden) and surgical excision of the salivary glands. Treatment usage was described via several summary statistics, including the mean and median time until any treatment, an anticholinergic claim, or a BoNT claim. Additionally, the number of patients with concurrent anticholinergic and BoNT use was estimated along with the mean and median number of days of overlap. This study also examined the number of different toxins used and the first, second, and third treatment types. The number of patients who received each type of treatment was assessed during both baseline and follow-up periods.

For patients treated with incobotulinumtoxinA, three utilization metrics were summarized: the number of days with BoNT injections during follow-up, the average number of weeks between injections, and evidence of switching to a non-incobotulinumtoxinA botulinum treatment. Additionally, the number of each provider type administering incobotulinumtoxinA injections was summarized.

### 5.7. Statistical Analyses

Analyses were conducted using the Aetion Evidence Platform (Aetion, Inc., New York, NY, USA) [15]. This analysis was not comparative. Descriptive analyses were conducted separately for the primary and secondary objectives and their respective cohorts. Continuous patient characteristics were described using mean, standard deviation (SD), median, and interquartile range (IQR), while categorical characteristics were presented as counts and percentages. Missing data were quantified, but not imputed, with patients assigned to unknown categories. Patients without evidence of a treatment or diagnosis were assumed to not have that specific treatment or diagnosis. The number of injections was summarized as a rate, calculated as the number of injections divided by person-time, and expressed as the number of injections per year at the patient level (e.g., Patient A had four injections per year during follow-up). The mean (SD) and median (IQR) numbers of injections per patient per year were presented at the cohort level (e.g., on average, men had 3 injections per year). The average time between injections was summarized as the mean number of weeks between injections at the patient level (e.g., Patient A had an average of 15 weeks between injections) and aggregated to present the mean (SD) and median (IQR) average number of months between injections at the cohort level (e.g., on average, men had a mean of 20 weeks between injections). Switching was presented as the count and proportions of patients who had evidence of a second treatment during follow-up that differed from their first recorded treatment.

For the secondary objective, a sensitivity analysis was conducted for incobotulinumtoxinA-treated patients to evaluate utilization metrics with an injection cadence aligned with guidelines of BoNT usage, where patients with multiple incobotulinumtoxinA claims less than 4 weeks apart were excluded, and follow-up ended 16 weeks after the final injection before a 32-week gap.

## Figures and Tables

**Table 1:** Characteristics of patients with sialorrhea.

	Overall Sialorrhea	Pediatric Sialorrhea	Adult Sialorrhea	IncobotulinumtoxinA Users
	N = 4073	N = 398	N = 3675	N = 177
**Age (years)**				
Mean (SD)	59.64 (23.09)	9.39 (4.64)	65.09 (16.89)	68.97 (17.80)
Median [IQR]	67.00 [50.00, 77.00]	10.00 [5.00, 13.00]	68.00 [56.00, 78.00]	73.00 [65.50, 80.50]
**Age Group, n (%)**				
2–17 years	398 (9.8%)	398 (100.0%)		6 (3.4%)
18–44 years	465 (11.4%)		465 (12.7%)	10 (5.6%)
45–64 years	1013 (24.9%)		1013 (27.6%)	27 (15.3%)
65+ years	2197 (53.9%)		2197 (59.8%)	134 (75.7%)
**Sex, n (%)**				
Male	2223 (54.6%)	237 (59.5%)	1986 (54.0%)	110 (62.1%)
Female	1850 (45.4%)	161 (40.5%)	1689 (46.0%)	67 (37.9%)
**Race, n (%)**				
Non-Hispanic White	2739 (67.2%)	259 (65.1%)	2480 (67.5%)	130 (73.4%)
Non-Hispanic Black	557 (13.7%)	24 (6.0%)	533 (14.5%)	15 (8.5%)
Non-Hispanic Asian	114 (2.8%)	18 (4.5%)	96 (2.6%)	9 (5.1%)
Hispanic	353 (8.7%)	29 (7.3%)	324 (8.8%)	15 (8.5%)
Unknown/missing	310 (7.6%)	68 (17.1%)	242 (6.6%)	8 (4.5%)
**Region, n (%)**				
Northeast	512 (12.6%)	29 (7.3%)	483 (13.1%)	27 (15.3%)
Midwest	974 (23.9%)	157 (39.4%)	817 (22.2%)	30 (16.9%)
South	1721 (42.3%)	149 (37.4%)	1572 (42.8%)	64 (36.2%)
West	861 (21.1%)	60 (15.1%)	801 (21.8%)	56 (31.6%)
Other	5 (0.1%)	3 (0.8%)	2 (0.1%)	0 (0.0%)
**Insurance Type, n (%)**				
Commercial only	1469 (36.1%)	398 (100.0%)	1071 (29.1%)	39 (22.0%)
Medicare only	2600 (63.8%)	0 (0.0%)	2600 (70.7%)	138 (78.0%)
Other	N < 5	N < 5	N < 5	N < 5

**Table 2:** Clinical characteristics and treatments at baseline and follow-up for patients with sialorrhea.

	Overall Sialorrhea		Pediatric Sialorrhea		Adult Sialorrhea		IncobotulinumtoxinA Users	
	N = 4073		N = 398		N = 3675		N = 177	
	Baseline	Follow-Up	Baseline	Follow-Up	Baseline	Follow-Up	Baseline	Follow-Up
**Comorbidities, n (%)**								
Alzheimer’s disease/dementia	358 (8.8%)	624 (15.3%)	N < 5	N < 5	357 (9.7%)	623 (17.0%)	11 (6.2%)	25 (14.1%)
Amyotrophic lateral sclerosis	245 (6.0%)	283 (6.9%)	N < 5	N < 5	245 (6.7%)	282 (7.7%)	8 (4.5%)	9 (5.1%)
Cerebral Palsy	328 (8.1%)	410 (10.1%)	223 (56.0%)	269 (67.6%)	105 (2.9%)	141 (3.8%)	7 (4.0%)	9 (5.1%)
Multiple sclerosis	62 (1.5%)	84 (2.1%)	N < 5	N < 5	61 (1.7%)	82 (2.2%)	N < 5	N < 5
Parkinson’s disease	710 (17.4%)	957 (23.5%)	N < 5	N < 5	710 (19.3%)	955 (26.0%)	110 (62.1%)	115 (65.0%)
Stroke	430 (10.6%)	655 (16.1%)	12 (3.0%)	21 (5.3%)	418 (11.4%)	634 (17.3%)	15 (8.5%)	29 (16.4%)
Traumatic brain injury	87 (2.1%)	176 (4.3%)	9 (2.3%)	18 (4.5%)	78 (2.1%)	158 (4.3%)	N < 5	9 (5.1%)
**Clinical Symptoms, n (%)**								
Anxiety	848 (20.8%)	1402 (34.4%)	22 (5.5%)	52 (13.1%)	826 (22.5%)	1350 (36.7%)	27 (15.3%)	39 (22.0%)
Depression	815 (20.0%)	1338 (32.9%)	6 (1.5%)	10 (2.5%)	809 (22.0%)	1328 (36.1%)	40 (22.6%)	54 (30.5%)
Dehydration	385 (9.5%)	916 (22.5%)	27 (6.8%)	77 (19.3%)	358 (9.7%)	839 (22.8%)	N < 5	37 (20.9%)
Dysphagia	1482 (36.4%)	2198 (54.0%)	141 (35.4%)	206 (51.8%)	1341 (36.5%)	1992 (54.2%)	66 (37.3%)	94 (53.1%)
Speech impairment	222 (5.5%)	393 (9.6%)	26 (6.5%)	51 (12.8%)	196 (5.3%)	342 (9.3%)	7 (4.0%)	11 (6.2%)
**incobotulinumtoxinA Indications, n (%)**								
Blepharospasm	53 (1.3%)	95 (2.3%)	N < 5	N < 5	52 (1.4%)	93 (2.5%)	11 (6.2%)	21 (11.9%)
Cervical Dystonia	91 (2.2%)	180 (4.4%)	5 (1.3%)	12 (3.0%)	86 (2.3%)	168 (4.6%)	15 (8.5%)	23 (13.0%)
Upper Limb Spasticity	153 (3.8%)	288 (7.1%)	78 (19.6%)	148 (37.2%)	75 (2.0%)	140 (3.8%)	8 (4.5%)	13 (7.3%)
**Anticholinergic Therapy, n (%)**								
Any anticholinergic	1525 (37.4%)	2827 (69.4%)	195 (49.0%)	340 (85.4%)	1330 (36.2%)	2487 (67.7%)	27 (15.3%)	25 (14.1%)
Glycopyrrolate	580 (14.2%)	1332 (32.7%)	149 (37.4%)	292 (73.4%)	431 (11.7%)	1040 (28.3%)	16 (9.0%)	14 (7.9%)
Benztropine	407 (10.0%)	477 (11.7%)	6 (1.5%)	12 (3.0%)	401 (10.9%)	465 (12.7%)	N < 5	N < 5
Scopolamine	617 (15.1%)	1308 (32.1%)	49 (12.3%)	89 (22.4%)	568 (15.5%)	1219 (33.2%)	11 (6.2%)	12 (6.8%)
Tropicamide	N < 5	N < 5	N < 5	N < 5	N < 5	N < 5	N < 5	N < 5
Biperiden	N < 5	N < 5	N < 5	N < 5	N < 5	N < 5	N < 5	N < 5
**Botulinum Toxin Therapy, n (%)**								
Any BoNT	306 (7.5%)	1389 (34.1%)	50 (12.6%)	152 (38.2%)	256 (7.0%)	1237 (33.7%)	46 (26.0%)	177 (100.0%)
incobotulinumtoxinA	9 (0.2%)	92 (2.3%)	N < 5	6 (1.5%)	7 (0.2%)	86 (2.3%)	N < 5	177 (100.0%)
rimabotulinumtoxinB	18 (0.4%)	529 (13.0%)	4 (1.0%)	11 (2.8%)	14 (0.4%)	518 (14.1%)	26 (14.7%)	27 (15.3%)
onabotulinumtoxinA	270 (6.6%)	894 (21.9%)	42 (10.6%)	133 (33.4%)	228 (6.2%)	761 (20.7%)	20 (11.3%)	15 (8.5%)
abobotulinumtoxinA	10 (0.2%)	36 (0.9%)	N < 5	13 (3.3%)	8 (0.2%)	23 (0.6%)	N < 5	N < 5

Note: Some cells are suppressed due to small counts.

**Table 3:** Treatment patterns in the sialorrhea population.

	Overall Sialorrhea	Pediatric Sialorrhea	Adult Sialorrhea
	N = 4073	N = 398	N = 3675
**First Treatment, n (%)**			
Patients with a treatment claim in follow-up	3241	366	2875
Anticholinergics	2013 (62.1%)	275 (75.1%)	1738 (75.1%)
BoNT monotherapy	1155 (35.6%)	77 (21.0%)	1078 (37.5%)
incobotulinumtoxinA	53 (1.6%)		51 (0.5%)
rimabotulinumtoxinB	428 (13.2%)		422 (1.6%)
onabotulinumtoxinA	653 (20.1%)		588 (17.8%)
abobotulinumtoxinA	21 (0.6%)		17 (1.1%)
Other combinations	73 (2.3%)	14 (3.8%)	59 (3.8%)
**Second Treatment, n (%)**			
Patients with a second treatment claim in follow-up	1270	201	1069
Anticholinergics	689 (54.3%)	116 (57.7%)	573 (53.6%)
BoNT monotherapy	468 (36.9%)	53 (26.4%)	415 (38.8%)
incobotulinumtoxinA	21 (1.7%)		19 (1.8%)
rimabotulinumtoxinB	179 (14.1%)		173 (16.2%)
onabotulinumtoxinA	255 (20.1%)		214 (20.0%)
abobotulinumtoxinA	13 (1.0%)		9 (0.8%)
Other combinations	113 (8.9%)	32 (15.9%)	81 (7.6%)
**Third Treatment, n (%)**			
Patients with a third treatment claim in follow-up	661	134	527
Anticholinergics	362 (54.8%)	82 (61.2%)	280 (53.1%)
BoNT monotherapy	240 (36.3%)	34 (25.4%)	206 (39.1%)
incobotulinumtoxinA	11 (1.7%)		
rimabotulinumtoxinB	94 (14.2%)		
onabotulinumtoxinA	129 (19.5%)		
abobotulinumtoxinA	6 (0.9%)		
Other combinations	59 (8.9%)	18 (13.4%)	41 (7.8%)
**Time until First Treatment in Follow-Up, days**			
Patients with any treatment claim	3874	391	3483
mean (SD) until any treatment	17.23 (88.00)	13.25 (28.58)	17.68 (92.31)
median [IQR] until any treatment	3.00 [0.50, 16.00]	4.00 [0.50, 18.00]	2.00 [0.50, 16.00]
Patients with an anticholinergic claim	2827	340	2487
mean (SD) until anticholinergic claim	37.42 (172.07)	52.25 (259.49)	35.39 (156.34)
median [IQR] until anticholinergic claim	6.00 [0.50, 20.00]	6.00 [0.50, 20.75]	6.00 [0.50, 20.00]
Patients with a BoNT claim	1389	152	1237
mean (SD) until BoNT claim	58.75 (224.86)	187.29 (359.36)	42.95 (196.78)
median [IQR] until BoNT claim	0.50 [0.50, 20.00]	31.00 [0.50, 154.00]	0.50 [0.50, 16.00]
Patients with surgical excision	13	N < 5	12
mean (SD) until surgical excision	5.88 (9.17)		3.96 (6.25)
median [IQR] until surgical excision	0.50 [0.50, 12.50]		0.50 [0.50, 7.75]
**Combination Therapy**			
Patients with evidence of concurrent anticholinergic and BoNT use, n (%)	241 (5.9%)	73 (18.3%)	168 (4.6%)
mean (SD) number of days of overlap	184.56 (230.50)	226.79 (261.39)	166.21 (213.96)
median [IQR] number days of overlap	113.00 [59.00, 215.50]	133.00 [73.50, 264.50]	101.00 [55.25, 196.75]
**Switching**			
Patients with an anticholinergic treatment claim in follow-up	2827	340	2487
Proportion of patients who switch to BoNT therapy, n (%)	309 (10.9%)	88 (25.9%)	221 (8.9%)
Patients with a BoNT claim in follow-up	1389	152	1237
Proportion of patients who switch to anticholinergic; n (%)	282 (20.3%)	92 (60.5%)	190 (15.4%)
**Number of Different Toxins, n (%)**			
Zero	2684 (65.9%)	246 (61.8%)	2438 (66.3%)
One	1233 (30.3%)	141 (35.4%)	1092 (29.7%)
Two or more	156 (3.8%)	11 (2.8%)	145 (3.9%)

BoNT, botulinum toxin; IQR, interquartile range; SD, standard deviation. Note: Some cells are suppressed due to small counts. The use of anticholinergics was observed in 15.3% of patients during the baseline period compared to 14.1% in the follow-up period (following incobotulinumtoxinA administration). Glycopyrrolate (59.3% to 56%) and scopolamine (40.7% to 48.0%) were the most commonly prescribed anticholinergics, similar to the overall sialorrhea population.

**Table 4:** IncobotulinumtoxinA utilization among patients with sialorrhea.

	IncobotulinumtoxinA Users	Sensitivity Cohort (Excluding Patients with Claims <4 or >32 Weeks Apart)
	N = 177	N = 153
**Follow-Up, Years**		
Mean (SD)	1.65 (1.75)	0.54 (0.51)
Median [IQR]	1.01 [0.48, 2.37]	0.31 [0.31, 0.63]
**Number of Injections**		
Mean number of injections per person (SD)	2.46 (2.63)	1.99 (1.80)
Median number of injections per person [IQR]	1.00 [1.00, 3.00]	1.00 [1.00, 2.00]
Mean number of injections per person-year (SD)	1.47 (1.08)	1.73 (1.00)
Median number of injections per person-year [IQR]	1.00 [0.80, 2.00]	1.00 [1.00, 2.00]
**Weeks between Injections**		
Number of patients with more than 1 injection	87 (49.2%)	65 (42.5%)
Mean number of weeks between injections per person (SD)	19.86 (15.09)	15.66 (4.10)
Median number of weeks between injections per person [IQR]	15.00 [13.00, 20.00]	14.14 [13.00, 17.14]
**Time until Treatment Switch, days**		
number of patients with a switch to non-incobotulinumtoxinA toxin	40	19
mean (SD)	219.35 (255.09)	86.05 (78.51)
median [IQR]	122.00 [85.50, 250.25]	84.00 [0.50, 116.00]
**Switching (n, %)**		
Patients with an administration of a non-incobotulinumtoxinA treatment during follow-up	40 (22.6%)	19 (12.4%)
onabotulinumtoxinA use	15 (8.5%)	N < 5
rimabotulinumtoxinB use	27 (15.3%)	15 (9.8%)
abobotulinumtoxinA use	N < 5	N < 5
**incobotulinumtoxinA Treatment Time (until switch or discontinuation), days**		
Mean number of weeks (SD)	187.22 (212.80)	175.35 (170.94)
Median number of weeks [IQR]	113.00 [99.00, 226.00]	113.00 [91.00, 219.00]
**Provider Types Administering incobotulinumtoxinA**		
Total number of injection events	435	305
Number of injection events administered by general/family medicine, n (%)	49 (11.3%)	48 (15.7%)
Number of injection events administered by neurology, n (%)	249 (57.2%)	186 (61.0%)
Number of injection events administered by physical medicine and rehabilitation specialist, n (%)	7 (1.6%)	7 (2.3%)
Number of injection events administered by ear, nose, and throat, n (%)	12 (2.8%)	7 (2.3%)
Number of injection events administered by psychiatry, n (%)	22 (5.1%)	22 (7.2%)
Number of injection events administered by other provider type; n (%)	96 (22.1%)	35 (11.5%)

Note: Some cells are suppressed due to small counts.

## Data Availability

Restrictions apply to the availability of these data. Data were obtained from Optum. For information on obtaining the underlying data used for this analysis, please contact Optum.
